# Validity of Routinely Collected Swedish Data in the International Enhanced Recovery After Surgery (ERAS) Database

**DOI:** 10.1007/s00268-021-06094-4

**Published:** 2021-04-07

**Authors:** Yin Xu, Ruzan Udumyan, Katja Fall, Olle Ljungqvist, Scott Montgomery, Ulf O Gustafsson

**Affiliations:** 1 Clinical Epidemiology and Biostatistics, School of Medical Sciences, Örebro University, Örebro, Sweden; 2 Department of Surgery, Örebro & Institute of Molecular Medicine and Surgery, Karolinska Institutet, Örebro University and University Hospital, Stockholm, Sweden; 3 Division of Surgery, Department of Clinical Sciences, Karolinska Institutet, Danderyd Hospital, 18288 Danderyd, Stockholm, Sweden; 4 Integrative Epidemiology, Institute of Environmental Medicine, Karolinska Institutet, 171 77 Stockholm, Sweden; 5 Clinical Epidemiology Division, Department of Medicine, Karolinska Institutet, Solna, Stockholm, Sweden; 6 Department of Epidemiology and Public Health, University College London, London, UK

## Abstract

**Background:**

This study aims to assess patient coverage, validity and data quality in the Swedish part of the International Enhanced Recovery After Surgery (ERAS) Interactive Audit System (EIAS).

**Method:**

All Swedish ERAS centers that recorded colorectal surgery data in EIAS between January 1, 2017, and December 31, 2017, were included (*N* = 12). Information registered in EIAS was compared with data from electronic medical records at each hospital to assess the overall coverage of EIAS. Twenty random-selected patients from each of the contributing centers were assessed for accuracy for a set of clinically relevant variables. All patients admitted to the contributing centers were included for the assessment of rate of missing on a selection of key clinical variables.

**Results:**

Eight hospitals provided complete information for the evaluation, while four hospitals only allowed assessment of coverage and missing data. The eight hospitals had an overall coverage of 98.8% in EIAS (*n* = 1301) and the four 86.7% (*n* = 811). The average agreement for the assessed postoperative outcome variables was 96.5%. The accuracy was excellent for ‘length of hospital stay,’ ‘reoperation,’ and ‘any complications,’ but lower for other types of complications. Only a few variables had more than 5% missing data, and missingness was associated with hospital type and size.

**Conclusion:**

This validation of the Swedish part of the international ERAS database suggests high patient coverage in EIAS and high agreement and limited missingness in clinically relevant variables. This validation approach or a modified version can be used for continued validation of the International ERAS database.

**Supplementary Information:**

The online version contains supplementary material available at 10.1007/s00268-021-06094-4.

## Introduction

Use of an evidence-based standardized protocol designed to optimize perioperative care in an enhanced recovery after surgery (ERAS) program results in faster recovery, reduced morbidity and shorter length of stay (LOS) in colorectal surgery [1]. In recent years, ERAS has spread to almost all operating specialties worldwide (www.erassociety.org). In order to receive continuous feedback on the quality of the clinical care, compliance to the ERAS protocol, perioperative data and outcome measures are consecutively recorded in the International ERAS® Interactive Audit System (EIAS) [2]. This database is used for implementation of the ERAS®Society Guidelines and to sustain these principles of care. EIAS currently contains more than 90,000 consecutively recorded patients, each with up to 300 recorded perioperative variables.

Since EIAS includes large numbers of patients with detailed perioperative data, the database constitutes a unique resource for research on patients undergoing colorectal surgery. The large sample of surgical patients who are managed in a controlled environment reflecting clinical reality, opens opportunities to study an array of yet unanswered clinical questions. However, the usefulness of a clinical database relies on the completeness and quality of the reported data. Therefore, the data needs to be validated to assess the potential for systematic errors, which can cause bias in study conclusions.

The aim of the study was to assess register coverage and data quality in terms of accuracy and completeness in the Swedish part of the international EIAS as well as to identify tools for future validations of the international EIAS.

## Material and methods

### Study population

All Swedish ERAS centers recording elective colorectal surgery data in EIAS between January 1, 2017, and December 31, 2017 (*N* = 12), were eligible for inclusion and all agreed to take part in the validation. In centers participating in the ERAS collaboration, it is mandatory to include all patients undergoing elective major colorectal surgery. They allowed access to the hospitals' local medical records system to enable a comparison between recorded data in EIAS and hospital electronic medical records (EMR). EIAS is an online- and web-based resource, which allows centers in different countries to enter peri-, intra-, and postoperative patient data in order to facilitate standardized data collection on recovery after surgery across centers. Two nurses and one medical doctor were trained in the validation process and performed the validation on site for each hospital, respectively.

The Swedish part of the International EIAS database is approved by Swedish authorities.

### Design

The validation procedure was designed by the Department of Clinical Epidemiology and Biostatistics, Örebro University, Sweden. Three main validation parameters were used in the process:

#### Coverage

To assess the patient coverage in EIAS during the study period, the number of patients who underwent elective colorectal surgery identified in EIAS was compared with the number of patients identified through EMR.

#### Accuracy

To assess accuracy of data entry in EIAS during the study period, concordance between EIAS and EMR was evaluated by investigating 11 important perioperative variables using data from 20 randomly selected patients from each contributing hospital.

#### Missing values

To quantify the extent of missing data, we calculated the proportion of patients with missing data on the 11 pre-specified variables among all patients admitted to the contributing hospitals. We further investigated selected hospital-, patient-, and surgery-level variables as potential predictors of missingness.

### Data analysis

The patient coverage in EIAS was examined by calculating the proportion of eligible patients identified through the EMRs who were registered in EIAS.

The accuracy between EIAS and EMR was assessed for the 11 variables by calculating the percent agreement (the number of patients whose response from the EIAS was confirmed in the EMR divided by the total number of patients). For binary variables, we also calculated sensitivity, specificity, positive predictive value (PPV), and negative predictive value (NPV), treating EMR data as the gold standard. Sensitivity = the number of patients reported to be yes in both EIAS and EMR divided by the number of patients reported to be yes in EMR, specificity = the number of patients reported to be no in both EIAS and EMR divided by the number of patients reported to be no in EMR, PPV = the number of patients reported to be yes in both EIAS and EMR divided by the number of patients reported to be yes in EIAS, and NPV = the number of patients reported to be no in both EIAS and EMR divided by the number of patients reported to be no in EIAS.

For binary variables, we also estimated the area under the receiver operating characteristic curve (AUC) and Cohen’s kappa statistic since they are summary measures of the accuracy. Bias-corrected bootstrap 95% confidence interval based on 1000 bootstrap replications was also reported. AUC and Cohen’s kappa were calculated for variables with dichotomous responses present in both data sources. AUC classifies values between 0.50 and 0.70 as poor, 0.70–0.80 as acceptable, 0.80–0.90 as excellent, and > 0.90 as outstanding [3]. Cohen’s kappa classifies values < 0 as indicating no agreement and 0–0.20 as slight, 0.21–0.40 as fair, 0.41–0.60 as moderate, 0.61–0.80 as substantial, and 0.81–1 as almost perfect agreement [4]. For length of stay, the individual intraclass correlation coefficient from one-way random effects model was reported.

Finally, we calculated the proportion of missing information for the 11 pre-specified variables in EIAS, and used *x*^2^ test or Fisher’s exact test (for variables with an expected cell size less than 5) to test whether the missingness was related to nine hospital-, patient-, and surgery-levels variables, including coverage (whether equals to 100%), length of stay (< 7 or ≥ 7 days), bed numbers (< 350 or ≥ 350), hospital type (academic or not), admission period (Jan–Mar, Apr–Jun, Jul–Sep, and Oct–Dec), sex, age (0–25, 26–50, 51–75, and 76–100), surgical type (open, laparoscopic, robotic, and through stoma), and procedure type (rectal or colonic and small bowel procedures). These variables were chosen based on their availability in EIAS, low proportions of missing data, and clinical importance.

Stata version 16.0 (StataCorp, College Station, Texas, United States of America) was used for statistical analysis.

## Results

Of the 12 eligible hospitals, 4 hospitals did not have the conversion key to link the EIAS records with the EMR and hence could not be included in the data accuracy evaluation. However, they were included in the evaluation of patient coverage and missing data.

### Coverage

Comparison of the EIAS records from 12 hospitals (*n* = 2112) with EMRs identified 151 patients omitted resulting in an overall coverage of 93.3%. The patient coverage in EIAS was above 96% for all eight hospitals that had the conversion key (Table 1) with the average coverage of 98.8%. The patient coverage among the four hospitals was 86.7%.

**Table 1:** Patients’ coverage in the International ERAS® Interactive Audit System (EIAS) database stratified by hospital

No	Unit name	Number of patients registered in the EIAS	Number of patients not registered in the EIAS	Coverage, %	Coverage^a^, %
*Hospitals with converting key*					
1	Ersta Hospital	198	3	98.5	98.5
2	Lidköping Hospital	93	0	100.0	100.0
3	Karlstad Hospital	198	5	97.5	98.0
4	St. Göran Hospital	166	0	100.0	100.0
5	NUS Umeå	100	4	96.2	97.1
6	Skövde Hospital	126	0	100.0	100.0
7	Danderyd Hospital	269	7	97.5	100.0
8	Örebro Hospital	151	8	96.0	96.2
	Total	1301	27	98.0	98.8
*Hospitals without converting key*					
1	Västerås Hospital	88	8	91.7	–
2	Östersund Hospital	63	21	75.0	–
3	Sahlgrenska Östra Hospital	441	95	82.3	–
4	NU–Sjukvården Näl Hospital	219	0	100.0	–
	Total	811	124	86.7	–

Some hospitals did not register in EIAS certain elective colorectal surgeries due to hospital-specific inclusion/exclusion criteria (small bowel resection (one case for Karlstad hospital, one case for NUS Umeå, and one case for Danderyd Hospital), patients with iliopsoas abscess (one case for Örebro Hospital), and patients with rectal prolapse (one case for Örebro Hospital) were not registered. Danderyd Hospital did not record reversal of Hartman or other stoma down procedures (*n* = 6).)

*ERAS *Enhanced recovery after surgery

^a^Coverage rate when patients omitted due to hospital-specific exclusion criteria are not counted

### Accuracy for LOS, overall complications, date of death, and reoperations

In the data from the 8 hospitals with a conversion key, the average overall data accuracy was 96.5%. High accuracy (91.25%) specificity (96.2%), PPV (91.8%), and NPV (91.00%) were observed for the variable measuring any complication at all during primary stay with sensitivity of 81.8% indicating acceptable agreement, Cohen’s Kappa of 0.80 indicating substantial agreement, and AUC of 0.89 suggesting excellent agreement (Table 2). Of 55 patients with complications recorded in the EMR, 45 could also be identified by EIAS. Another 10 patients had been misclassified as free of complications and yet another 4 complication-free patients in EMR have been misclassified as having complications in EIAS. High accuracy was observed for length of stay during primary stay (93.75%) and date of death (100.0%). Based on the intraclass correlation coefficient, the accuracy for length of stay during primary stay was good (0.84). High accuracy, sensitivity, specificity, PPV, and NPV were also observed for reoperations (all above 94%), with Cohen’s Kappa of 0.93 indicating near perfect agreement, and AUC of 0.97 suggesting outstanding agreement.

**Table 2:** Accuracy of selected variables related to the length of stay and complications during primary stay in 160 randomly selected patients undergoing elective colorectal surgery at 8 ERAS centers in Sweden in 2017

Variables	True positive	False positive	False negative	True negative	Sensitivity (%)	Specificity (%)	PPV (%)	NPV (%)	Accuracy (%)	Cohen’s kappa^a^ (95%CI)	AUC^b^ (95%CI)
Length of stay (nights)	–	–	–	–	–	–	–	–	93.75	0.84 (0.79, 0.88)	–
Complications at all	45	4	10	101	81.82	96.19	91.84	90.99	91.25	0.80 (0.69, 0.84)	0.89 (0.84, 0.94)
Date of death	–	–	–	–	–	–	–	–	100.00	–	–
Reoperations	16	1	1	142	94.12	99.30	94.12	99.30	98.75	0.93 (0.82, 1.00)	0.97 (0.91, 1.00)
Pneumonia	0	0	1	159	–	–	–	99.38	99.38	0.00 (0.00, 0.00)	–
Urinary tract infection	0	0	0	160	–	–	–	100.00	100.00	–	–
Intraperitoneal or retroperitoneal abscess	2	1	4	153	33.33	99.35	66.67	97.45	96.88	0.43 (−0.01, 0.85)	0.66 (0.46, 0.87)
Sepsis	2	0	8	150	20.00	100.00	100.00	94.94	95.00	0.32 (0.00, 0.66)	0.60 (0.47, 0.73)
Anastomotic leak	8	0	2	150	80.00	100.00	100.00	98.68	98.75	0.88 (0.66, 1.00)	0.90 (0.77, 1.00)
Mechanical bowel obstruction	0	1	1	158	0.00	99.37	0.00	99.37	98.75	−0.01 (−0.02, 0.00)	0.50 (0.00, 1.00)
Postoperative paralytic ileus	10	9	8	133	55.56	93.66	52.63	94.33	89.38	0.48 (0.26, 0.68)	0.75 (0.63, 0.87)

EMR data was treated as the gold standard. True positive is the number of patients reported to be yes in both EIAS and EMR, false positive is the number of patients reported to be yes in EIAS and no in EMR, false negative is the number of patients reported to be no in EIAS and yes in EMR, and true negative is the number of patients reported to be no in both EIAS and EMR. Sensitivity is the true positive rate (the number of patients reported to be yes in both EIAS and EMR divided by the number of patients reported to be yes in EMR), specificity is the true negative rate (the number of patients reported to be no in both EIAS and EMR divided by the number of patients reported to be no in EMR), PPV is defined as the number of patients reported to be yes in both EIAS and EMR divided by the number of patients reported to be yes in EIAS, NPV is defined as the number of patients reported to be no in both EIAS and EMR divided by the number of patients reported to be no in EIAS, and accuracy is the proportion of patients with accurately recorded value in EIAS (the number of patients whose response from EIAS was confirmed in EMR divided by the total number of patients). AUC and Cohen’s kappa were calculated for variables with dichotomous responses present in both data sources

*PPV *Positive predictive value; *NPV *Negative predictive value; *CI *Confidence interval; *AUC *Area under the receiver operating characteristic curve; *ERAS *Enhanced recovery after surgery; *EIAS *International ERAS Interactive audit system; *EMR *Electronic medical records

^a^For length of stay, the individual intraclass correlation coefficient from one-way random effects model was reported. Cohen’s kappa indicates values < 0 as indicating no agreement and 0–0.20 as slight, 0.21–0.40 as fair, 0.41–0.60 as moderate, 0.61–0.80 as substantial, and 0.81–1 as almost perfect agreement

^b^AUC indicates 0.5–0.7 as poor, 0.7–0.8 as acceptable, 0.8–0.9 as excellent, and > 0.9 as outstanding

### Accuracy for specific types of complications

When comparing complications separately, 6 out of 7 variables show an accuracy of over 95% between EIAS and EMR (Table 2) and of 89.38% for postoperative paralytic ileus. The specificity and NPV were over 93% for all categorical variables that are applicable. The sensitivity was 80.00% for anastomotic leak but was low for postoperative paralytic ileus (55.6%), abscess (33.3%), sepsis (20.0%), and mechanical bowel obstruction (0.0%), which was due to the low prevalence of these complications that had been reported in EMR. The PPV was 100% for sepsis and anastomotic leak, but was low for abscess (66.7%), postoperative paralytic ileus (52.6%), and mechanical bowel obstruction (0.0%), which was also due to the low prevalence of these complications that had been reported in EIAS.

Based on Cohen’s kappa coefficients, the agreement between EIAS and EMR was considered as near perfect for reoperation (0.93) and anastomotic leak (0.88). The accuracy was however moderate for abscess (0.43), and postoperative paralytic ileus (0.48), and fair (0.32) for sepsis. The agreement for pneumonia was slight (0), but this was due to only one single patient that had been misclassified as no pneumonia. The agreement for mechanical bowel obstruction was worse than what would be expected by chance (<0), but again this calculation is based on only two patients that both had been misclassified in EIAS as the opposite of the true value from EMR.

Based on AUC, the agreement between EIAS and EMR was considered outstanding for reoperations (0.97) and excellent for anastomotic leak (0.90). The accuracy was acceptable for postoperative paralytic ileus (0.75), but poor for abscess (0.66), sepsis (0.60), and mechanic bowel obstruction (0.50).

### Missing values

The proportion of missing values for the pre-specified variables in both hospitals with and without conversion key is presented in Table 3. The mean missing rate for the centers without conversion key was 2.74% and for the centers with conversion key 1.92%. In the latter group, no missing was observed for total IV volume of fluids day zero, complications during primary stay, or reoperations during primary stay. Most missing information was observed for termination of urinary drainage (8.69%) and intraoperative blood loss (5.84%). The high missingness was due to two hospitals, while the remaining had high agreement for these variables. The remaining six variables had 0.54–1.92% missing information.

**Table 3:** The frequency and percentage of missing values for selected variables from the International ERAS® Interactive Audit System (EIAS) among all patients included in the validation

Variables	Missing, *n* (%) in 8 centers with conversion key (*n* = 1301)	Missing, *n* (%) in 4 centers without conversion key (*n* = 811)	Missing, *n* (%) in all 12 centers (*n* = 2112)
Oral bowel preparation	7 (0.54)	1 (0.12)	8 (0.38)
Preoperative oral carbohydrate treatment	13 (1.00)	14 (1.73)	27 (1.28)
Intraoperative blood loss	76 (5.84)	14 (1.73)	90 (4.26)
Total IV volume of fluids day zero	0 (0.00)	3 (0.37)	3 (0.14)
Termination of urinary drainage	113 (8.69)	135 (16.65)	248 (11.74)
Complications during primary stay	0 (0.00)	3 (0.37)	3 (0.14)
Complications after primary stay	25 (1.92)	33 (4.07)	58 (2.75)
Length of stay during primary stay (nights)	10 (0.77)	8 (0.99)	18 (0.85)
Total length of stay (nights)	10 (0.77)	7 (0.86)	17 (0.80)
Reoperations during primary stay	0 (0.00)	4 (0.49)	4 (0.19)
Reoperations after primary stay	21 (1.61)	22 (2.71)	43 (2.04)
Complete case	216 (16.60)	177 (21.82)	393 (18.61)

Complete case was defined as a patient with information available for all variables listed here. The frequency and percentage of non-complete case was reported here

*ERAS *Enhanced recovery after surgery

### Predictors of missingness

Patients who underwent rectal procedures were statistically significantly more likely to have missing information for termination of urinary drainage compared with patients who underwent colonic or small bowel procedures (Supplementary Table 1). Non-academic hospitals, hospitals with a hospital bed number less than 350, female patients, and patients who underwent a colonic and bowel procedure were statistically significantly more likely to have missing values for intraoperative blood loss. Overall, academic hospitals and hospitals with beds numbering more than 350 reported more complete cases based on 11 pre-specified variables (Supplementary Table 1).

### Key validation variables

**Fig. 1:**
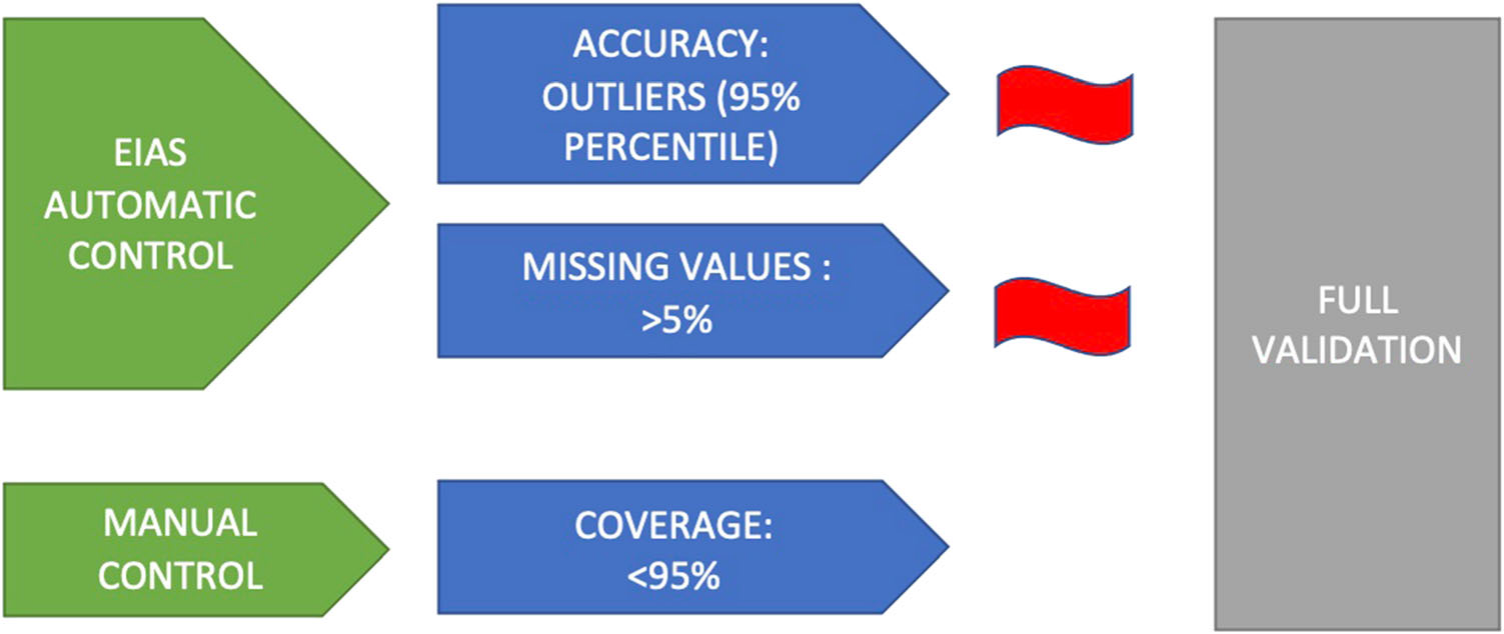
Alternative model for continuous validation of EIAS. We propose annual control for accuracy of the EIAS data. For Accuracy and Missing Values, the EIAS system itself can perform the checking annually and/or on demand: Accuracy: EIAS will automatically check for any deviation from average values in the EIAS system, and if outliers are outside the 95% percentile in any of the eleven accuracy variables a red warning flag will be raised. Missing Values: If more than 5% in any of the eleven variables are missing another red flag will be raised. Coverage: To signal and prevent non-participation, 20% of centers connected to EIAS will randomly be checked for coverage (by comparing data in EIAS and local operation planning systems). Low coverage < 90% or more than 4 red flags will result in a full validation according to the current study.

In order to find key variables that can be used for a faster validation process two important qualities were sought. First, the variable had to be valid and clinically important and secondly act as a proxy for the validity in other variables. Although the performance differed across centers, no clear patterns were observed between coverage, accuracy and missing values. However, based on the results in the current validation for the three main validation parameters separately, we suggest an alternative model that would be practically feasible to use for continuous validation of EIAS (Fig. 1).

## Discussion

The results from the current validation of the Swedish part of the international database indicate a high coverage of ERAS. The results further suggest a high accuracy for the majority, but not all, of the variables assessed. Only a few of the selected variables had more than 5% missing data, and the degree of missing data was partially associated with hospital type and size.

Several studies and meta-analysis have demonstrated that the ERAS program results in significant reduction in morbidity and length of stay [5, 6] in patients undergoing colorectal surgery compared to traditional perioperative care. However, the majority of data are reported from single center retrospective cohort studies or small RCTs. Since 2010, a growing number of ERAS centers globally register to the International ERAS Interactive Audit System (EIAS) [7]. Today the database contains more than 90,000 consecutively recorded colorectal surgery patients, each with up to 300 recorded variables. This is a unique resource for high-quality research that can facilitate progress and development of models for implementation of best perioperative practices, but its use has so far been limited [7]. Since high register coverage and data quality are crucial components in achieving high-quality research [8], validation will be instrumental.

In the current validation study, patient coverage was classified as good to excellent in all centers and well on par with other similar databases and national quality registries [9–12]. High coverage is an important quality marker indicating low probability of patients’ systematic omission (e.g., due to mortality or complications). Consequently, selection bias due to selective inclusion of patients into the database is minimized.

There are no set standards for how to define information agreement in validations, although 90–100% has been defined excellent and 80–89% as good in some studies [10]. By this standard, the accuracy of data for one of the most important variables, any complications, in the current study showed excellent agreement (91%), with a substantial Cohen’s kappa inter-rater reliability. The average overall agreement was excellent (96.5) for individual complications and other clinically important variables, such as reoperations and length of stay, while agreement expressed as Cohen’s kappa and AUC was lower for abscess, sepsis, mechanic bowel obstruction and pneumonia. Few specific events might, however, make it difficult to draw firm conclusions from these analyses. Still, a high rate of overall agreement is an important quality indicator, since colorectal surgery complications traditionally have been reported with a vast diversity and significant variations in complication rates.

Missing data are unavoidable in epidemiological and clinical studies with potential implications for the validity of the study. The proportion of missing values was relatively low for most considered variables. Although the largest proportion of missing values was observed for termination of urinary drainage and intraoperative blood loss, a large proportion of values missing in EIAS for these two variables were missing also in EMR. Furthermore, the high rate of missing values was largely caused by two centers only. Although withdrawal of urinary catheter is an important proxy variable for fast mobilization, the medical staff simply forget to register the catheter withdrawal, a problem that has been well known for some time. Also, it is well known that surgeons often forget to report intraoperative blood loss due to tiredness in the end of the operation. However, these issues underline the importance of validation in order to identify variables that need to either be improved or to be handled with care in future studies.

Further analysis revealed inter-hospital variation in data missingness and suggested that hospital type (academic vs non-academic) and size may influence data reporting.

To implement the current validation tool on all ERAS databases in different countries worldwide may seem like an overwhelming task. Therefore, and since the database needs continuous revalidation, access to simpler methods for validation would facilitate the process.

Although no clear patterns were observed between coverage, accuracy and missing values in important variables that may act as predictors for each other, we have used the data in the current study to suggest an alternative way to continuously validate data in EIAS (Fig. 1).

Some limitations of this validation study should be noted. First, only eight of the twelve hospitals could provide data for all parameters of the validation as the remaining four lacked a key enabling cross-linkage between hospital records and ERAS. However, on the hospitals that lacked the conversion key (25%), at least a validation on coverage and missing data could be performed, and with good results. Whether a high coverage in this group can tell anything of accuracy is however difficult to know. Nevertheless, this highlights an importance of securing that the conversion key is saved by all participating hospitals.

Secondly, the design of the validation model is new which means that it may have shortcomings that we might have difficulty correcting for. However, since there is no available gold standard design in how to conduct a validation of large medical databases, we launched this model to achieve the most relevant results. In contrast to other models [10–12]] the current validation has only checked accuracy and missing values for variables which have been assessed to be particularly important for clinical outcomes. This is of great importance for how to interpret data. The average agreement among selected variables that are regarded as clinically important will be more accurate than an average based on a mix of variables. Furthermore, the practical part of the validation was conducted on site by independent validators, which is a strength of this study. Other limitations are that the quality of source documentation (EMR) has not been validated in this study and that the findings may be subject to Type 2 Error considering the relatively low number of randomly selected patients in each center. In addition, a limited and selected number of variables registered in the database were used as proxies for the validity of the entire database. If the current choices are better than other variables to reflect the overall accuracy remains an open question. It can be argued that other variables should have been selected. For instance, surgical site infections (SSIs), considered an important outcome variable in colorectal surgery was not specifically included in the validation. Currently, it is one of many complications covered under the heading ‘Any complication.’

In conclusion, this validation of the Swedish part of the international ERAS database provides critical information for the development and adequate interpretation of multicenter studies in Sweden. The validation tool, or a modified version of it, can be used to validate other parts of the ERAS database to enable high-quality studies on an international scale.

## Supplementary Information

Below is the link to the electronic supplementary material.

Supplementary file1 (DOCX 23 KB)
