# Supplementary material for: Validity of Routinely Collected Swedish Data in the International Enhanced Recovery After Surgery (ERAS) Database
Source: World J Surg. 2021 Apr 7;45(6):1622–9. doi: 10.1007/s00268-021-06094-4 (PMC8093151; doi:10.1007/s00268-021-06094-4)
Supplement: Supplementary file 1 — Supplementary file1 (DOCX 23 KB) [file 268_2021_6094_MOESM1_ESM.docx]

Supplementary Table 1. The missing rate (%) for eight variables stratified by some hospital-, patient-, and surgical-levels variables

|  | Variables, n (%) | | | | | | | | |
| --- | --- | --- | --- | --- | --- | --- | --- | --- | --- |
|  | Oral  bowel  preparation | Preoperative  Oral  Carbohydrate  treatment | Intraoprative  blood  loss | Termination  Of urinary  drainage | Complications  after  primary  stay | Length  of stay  during  primary  stay | Total  length  of stay | Reoperation  after  primary  stay | Complete-case |
| Coverage |  |  |  |  |  |  |  |  |  |
| <100% | 0 (0.00) | 7 (1.08) | 7 (1.08) | 54 (8.35) | 15 (2.32) | 4 (0.62) | 4 (0.62) | 12 (1.85) | 79 (12.21) |
| =100% | 7 (1.07) | 6 (0.92) | 69 (10.55) | 59 (9.02) | 10 (1.53) | 6 (0.92) | 6 (0.92) | 9 (1.38) | 137 (20.95) |
| Unadjusted association | *p* = 0.015 | *p* = 0.766 | *p* < 0.001 | *p* = 0.665 | *p* = 0.300 | *p* = 0.753 | *p* = 0.753 | *p* = 0.493 | *p* < 0.001 |
| Length of stay |  |  |  |  |  |  |  |  |  |
| < 7 | 6 (0.73) | 8 (0.97) | 57 (6.94) | 52 (6.33) | 2 (0.24) | 0 (0.00) | 0 (0.00) | 1 (0.12) | 116 (14.13) |
| >= 7 | 1 (0.22) | 5 (1.08) | 19 (4.11) | 58 (12.55) | 13 (2.81) | 0 (0.00) | 0 (0.00) | 10 (2.16) | 90 (19.48) |
| Unadjusted association | *p* = 0.433 | *p* = 0.853 | *p* = 0.039 | *p* < 0.001 | *p* < 0.001 | *-* | *-* | *p* < 0.001 | *p* = 0.012 |
| Bed numbers |  |  |  |  |  |  |  |  |  |
| <350 | 0 (0.00) | 0 (0.00) | 50 (10.94) | 41 (8.97) | 8 (1.75) | 4 (0.88) | 4 (0.88) | 5 (1.09) | 94 (20.57) |
| >=350 | 7 (0.83) | 13 (1.54) | 26 (3.08) | 72 (8.53) | 17 (2.01) | 6 (0.71) | 6 (0.71) | 16 (1.90) | 122 (14.45) |
| Unadjusted association | *p* = 0.104 | *p* = 0.006 | *p* < 0.001 | *p* = 0.788 | *p* = 0.741 | *p* = 0.748 | *p* = 0.748 | *p* = 0.273 | *p* = 0.005 |
| Hospital Type |  |  |  |  |  |  |  |  |  |
| Non-academic | 2 (0.33) | 4 (0.65) | 61 (9.92) | 51 (8.29) | 12 (1.95) | 5 (0.81) | 5 (0.81) | 7 (1.14) | 121 (19.67) |
| Academic | 5 (0.73) | 9 (1.31) | 15 (2.19) | 62 (9.04) | 13 (1.90) | 5 (0.73) | 5 (0.73) | 14 (2.04) | 95 (13.85) |
| Unadjusted association | *p* = 0.457 | *p* = 0.274 | *p* < 0.001 | *p* = 0.634 | *p* = 0.941 | *p* = 0.862 | *p* = 0.862 | *p* = 0.197 | *p* = 0.005 |
| Registration period |  |  |  |  |  |  |  |  |  |
| Jan-Mar | 2 (0.68) | 0 (0.00) | 15 (5.10) | 29 (9.86) | 7 (2.38) | 2 (0.68) | 2 (0.68) | 8 (2.72) | 50 (17.01) |
| Apr-Jun | 0 (0.00) | 5 (1.52) | 24 (7.29) | 23 (6.99) | 2 (0.61) | 1 (0.30) | 1 (0.30) | 2 (0.61) | 53 (16.11) |
| Jul-Sep | 2 (0.63) | 2 (0.63) | 17 (5.40) | 33 (10.48) | 7 (2.22) | 3 (0.95) | 3 (0.95) | 6 (1.90) | 54 (17.14) |
| Oct-Dec | 3 (0.83) | 6 (1.65) | 20 (5.51) | 28 (7.71) | 9 (2.48) | 4 (1.10) | 4 (1.10) | 5 (1.38) | 59 (16.25) |
| Unadjusted association | *p* = 0.462 | *p* = 0.086 | *p* = 0.628 | *p* = 0.332 | *p* = 0.187 | *p* = 0.673 | *p* = 0.673 | *p* = 0.192 | *p* = 0.979 |
| Procedure type |  |  |  |  |  |  |  |  |  |
| Rectal | 6 (1.76) | 3 (0.88) | 10 (2.93) | 43 (12.61) | 9 (2.64) | 2 (0.59) | 2 (0.59) | 10 (2.93) | 65 (19.06) |
| Colonic and bowel | 1 (0.10) | 10 (1.04) | 66 (6.88) | 70 (7.29) | 16 (1.67) | 8 (0.83) | 8 (0.83) | 11 (1.15) | 151 (15.73) |
| Unadjusted association | *p* = 0.002 | *p* = 1.000 | *p* = 0.008 | *p* = 0.003 | *p* = 0.261 | *p* = 1.000 | *p* = 1.000 | *p* = 0.025 | *p* = 0.155 |
| Surgical type |  |  |  |  |  |  |  |  |  |
| Open | 1 (0.18) | 8 (1.41) | 25 (4.40) | 58 (10.21) | 13 (2.29) | 5 (0.88) | 5 (0.88) | 12 (2.11) | 98 (17.25) |
| Laparoscopic | 2 (0.37) | 3 (0.56) | 41 (7.66) | 38 (7.10) | 8 (1.50) | 5 (0.93) | 5 (0.93) | 6 (1.12) | 86 (16.07) |
| Robotic | 4 (2.40) | 2 (1.20) | 7 (4.19) | 17 (10.18) | 3 (1.80) | 0 (0.00) | 0 (0.00) | 3 (1.80) | 28 (16.77) |
| Through Stoma | 0 (0.00) | 0 (0.00) | 3 (9.68) | 0 (0.00) | 1 (3.23) | 0 (0.00) | 0 (0.00) | 0 (0.00) | 4 (12.90) |
| Unadjusted association | *p* = 0.030 | *p* = 0.497 | *p* = 0.059 | *p* = 0.070 | *p* = 0.542 | *p* = 0.750 | *p* = 0.750 | *p* = 0.567 | *p* = 0.898 |
| Age group |  |  |  |  |  |  |  |  |  |
| 0-25 | 0 (0.00) | 0 (0.00) | 3 (21.43) | 0 (0.00) | 0 (0.00) | 0 (0.00) | 0 (0.00) | 0 (0.00) | 3 (21.43) |
| 26-50 | 3 (1.99) | 3 (1.99) | 8 (5.30) | 8 (5.30) | 5 (3.31) | 3 (1.99) | 3 (1.99) | 5 (3.31) | 25 (16.56) |
| 51-75 | 3 (0.40) | 5 (0.67) | 42 (5.64) | 65 (8.72) | 13 (1.74) | 5 (0.67) | 5 (0.67) | 11 (1.48) | 116 (15.57) |
| 76-100 | 1 (0.26) | 5 (1.28) | 23 (5.90) | 40 (10.26) | 7 (1.79) | 2 (0.51) | 2 (0.51) | 5 (1.28) | 72 (18.46) |
| Unadjusted association | *p* = 0.127 | *p* = 0.321 | *p* = 0.152 | *p* = 0.232 | *p* = 0.548 | *p* = 0.292 | *p* = 0.292 | *p* = 0.383 | *p* = 0.559 |
| Sex |  |  |  |  |  |  |  |  |  |
| Male | 4 (0.60) | 8 (1.20) | 27 (4.06) | 66 (9.92) | 14 (2.11) | 5 (0.75) | 5 (0.75) | 13 (1.95) | 110 (16.54) |
| Female | 3 (0.47) | 5 (0.79) | 49 (7.70) | 47 (7.39) | 11 (1.73) | 5 (0.79) | 5 (0.79) | 8 (1.26) | 106 (16.67) |
| Unadjusted association | *p* = 1.000 | *p* = 0.450 | *p* = 0.005 | *p* = 0.105 | *p* = 0.622 | *p* = 0.944 | *p* = 0.944 | *p* = 0.319 | *p* = 0.952 |

Note. Total IV volume of fluids day zero, complications during primary stay, and reoperations during primary stay were not included since there were no missing values. Pearson’s *x*^2^ test or Fisher exact test (for categorical variables with cell size less than 5) was performed to test the unadjusted association between center and missing for each variables listed here. Complete case was defined as a patient with information available for all variables listed here. The frequency and percentage of non-complete case was reported here.
